# Central Obesity and *H*. *pylori* Infection Influence Risk of Barrett’s Esophagus in an Asian Population

**DOI:** 10.1371/journal.pone.0167815

**Published:** 2016-12-09

**Authors:** Chih-Cheng Chen, Yao-Chun Hsu, Ching-Tai Lee, Chia-Chang Hsu, Chi-Ming Tai, Wen-Lun Wang, Cheng-Hao Tseng, Chao-Tien Hsu, Jaw-Town Lin, Chi-Yang Chang

**Affiliations:** 1 Department of Internal Medicine, E-Da Hospital, I-Shou University, Kaohsiung, Taiwan; 2 Department of Pathology, E-Da Hospital, I-Shou University, Kaohsiung, Taiwan; 3 School of Medicine and Big Data Research Centre, Fu Jen Catholic University, New Taipei City, Taiwan; Northwestern University Feinberg School of Medicine, UNITED STATES

## Abstract

**Background and Aim:**

The prevalence rates of Barrett’s esophagus (BE) in western countries are higher than Asian ones, but little is known about their difference among risk factors of BE. The aim of this study is to investigate the associations of various risk factors including central obesity, body mass index (BMI), metabolic syndrome and *H*. *pylori* infection, with BE.

**Methods:**

A total of 161 subjects with BE were enrolled and compared to age- and gender-matched controls randomly sampled (1:4) from check-up center in same hospital. Central obesity was defined by waist circumference (female>80cm; male>90cm), metabolic syndrome by the modified National Cholesterol Education Program Adult Treatment Panel III criteria in Taiwan. Independent risk factors for BE were identified by multiple logistic regression analyses.

**Results:**

The mean age for BE was 53.8±13.7 years and 75.8% was male. *H*. *pylori* infection status was detected by the rapid urease test with the prevalence of 28.4% and 44.4% in the BE patients and controls, respectively. The univariate logistic regression analyses showed the risk was associated with higher waist circumference (odds ratio [OR], 2.53; 95% confidence interval [CI], 1.78–3.60), metabolic syndrome (OR, 2.02; 95% CI, 1.38–2.96) and negative *H*. *pylori* infection (OR, 0.50; 95% CI, 0.34–0.74). However, multivariate logistic regression analyses revealed that BE associated with higher waist circumference (adjusted OR, 2.79; 95% CI, 1.89–4.12) and negative *H*. *pylori* infection (adjusted OR, 0.46; 95% CI, 0.30–0.70).

**Conclusions:**

Central obesity is associated with a higher risk of BE whereas *H*. *pylori* infection with a lower risk in an ethnic Chinese population.

## Introductions

Barrett’s esophagus (BE), defined as specialized intestinal metaplasia involving more than 1 cm above the esophagogastric junction [1], is an established precancerous lesion of esophageal adenocarcinoma [2]

The prevalence of BE is higher in the West than in Asian countries [3]. Population-based studies by Ronkainen et al.[4] and Zagari et al.[5] reported that the prevalence was around 1.3–1.6% in Western countries. Even more, a prevalence up to 6.8% was reported among subjects receiving screening colonoscopies in the United States [6]. On the contrary, large-scale studies from Chinese populations [7, 8] showed a prevalence of 0.2–1.0%. Moreover, short-segment BE (<3cm in length) accounts for 75.6~81.5% of the cases. Besides, while esophageal adenocarcinoma has overpassed squamous cell carcinoma in the West, it remains rare in the East [9].

Difference in the prevalence may arise from distinct risk components between Western and Asian populations. In a recent meta-analysis that pooled 51 studies, Shiota and colleagues showed that risk factors of BE in Asia were similar to those seen in Western countries and included reflux symptoms, male sex, hiatus hernia, and smoking. [3] Intriguingly, they found no association with obesity or *Helicobacter pylori* infection, but heterogeneous methodology across studies precluded a firm conclusion. We can see from 4 studies in Asia[10–13], elevated waist circumference, rather than BMI (defined as > 25kg/m^2^), was more associated with histologic BE.

The aim of this study was to clarify the risk factors for BE in an Asian population, with a particular focus on central obesity, body mass index (BMI), metabolic syndrome, and *H*. *pylori* infection.

## Materials and Methods

### Study design and participants

This was a hospital-based matched case-control study. Subjects undergoing endoscopic survey for various upper GI symptoms or health check-up were prospectively screened for the presence of BE. For those with visible columnar-type epithelium proximal to the gastroesophageal folds, standardized endoscopic biopsy protocol (random biopsy from four quadrants, every 2cm) were performed[14]. This study standardized the detection method in all participants with the rapid urease test (ASAN Helicobacter Test, ASAN Pharmaceuticals, Co., Ltd, Korea) examining gastric mucosa routinely taken from the antrum. BE was defined as histologically proven specialized intestinal metaplasia. The following patients were excluded: patients took proton-pump inhibitors (PPI) in recent two weeks, patients with acute upper gastrointestinal bleeding (both ulcer and variceal bleeding), patients with other critical illnesses that could not complete risk factor survey, or patients with underlying malignancy.

The controls were healthy individuals who received EGD for routine health check-up with neither erosive esophagitis nor Barrett’s esophagus on examination. They were randomly sampled from the database of health check-up center in the same enrollment period to match 4:1 with BE cases by age and gender.

Demographic data were recorded including age, gender, BMI, obesity (BMI ≥ 27kg/m^2^, according to the Ministry of Health and Welfare in Taiwan[15]), waist circumference, central obesity, alcohol use, smoking, metabolic syndrome, erosive esophagitis, hiatal hernia, and gastroesophageal reflux symptoms. We also recorded the EGD findings and their reflux disease questionnaire scores[16]. Central obesity was defined by waist circumference (female>80cm; male>90cm)[17]. Metabolic syndrome was documented by the modified National Cholesterol Education Program (NCEP) Adult Treatment Panel III criteria in Taiwan[18]. *H*. *pylori* infection was defined by positive CLO test. This study was approved by the Institutional Review Board of E-Da Hospital and I-Shou University with IRB No. EMRP-099-017. Written Informed consents were obtained in all BE case group and approved by our ethics committee.

### Statistical analysis

For demographic characteristics, continuous variables were compared by t-test and the categorical variables were analyzed by either χ^2^-test or Fisher’s exact test. Univariate logistic regression analyses were repeated to explore potential risk factors for BE. Multivariate-adjusted logistic regression analysis was then performed to identify independent risk factors. Odds ratio (ORs) along with their 95% confidence intervals (CIs) were estimated for each variable. All analyses were conducted using SAS, version 9.1 (SAS Institute, Cary, NC), with a *P* value less than 0.05 considered as statistically significant.

## Results

### Study population

We totally enrolled 161 BE cases and 644 age- and gender-matched controls from February 2007 to December 2010. The demographic characteristics were summarized in Table 1. The mean (±standard deviation) age of our BE cases was 53.8±13.7 years, and 75.8% of them were male. As compared with their controls, BE cases seemed to have higher BMI (25.2 ± 3.6 kg/m^2^ vs. 24.3 ± 3.5 kg/m^2^, p = 0.006), waist circumference (87.5 ± 10.1 cm vs. 82.7 ± 10.0 cm, p < 0.001), and prevalence of metabolic syndrome (41% vs. 24.1%), but less *H*. *pylori* infection (28.4% vs. 44.4%, p < 0.001). Only 60.2% of these BE patients had erosive esophagitis and 64.6% of them reported reflux symptoms.

**Table 1:** Demographic characteristics of BE cases and controls.

Characteristic	BE	Control	*p*-value
	(n = 161)	(n = 644)	
**Age(yrs) (mean ± s.d.)**	53.8±13.7	53.7±13.6	0.95
**Sex (male) (n, %)**	122 (75.8)	488 (75.8)	1.0
**BMI (kg/m**^**2**^**) (mean ± s.d.)**	25.2±3.6	24.3±3.5	0.006*
**Obesity (BMI≥ 27) (n, %)**	42(26.1)	135 (21.0)	0.16
**Waist (cm) (mean ± s.d.)**	87.5±10.1	82.7±10.0	<0.001*
**Central Obesity (n, %)**	84 (52.2)	189 (29.3)	<0.001*
**Alcohol use (n, %)**	61 (37.9)	213 (33.1)	0.25
**Smoking (n, %)**	46 (28.6)	192 (29.8)	0.76
**MetS (%)**	32/78 (41.0)	155 (24.1)	0.05*
**ERD (n,%)**	97 (60.2)	0 (0)	<0.001
**Hiatal hernia (n, %)**	17 (10.6)	0 (0)	<0.001
**GERD symptoms**	104 (64.6)	NA	
**H. pylori infection (n, %)**	42/148 (28.4)	261/588(44.4)	<0.001

BE, Barrett's esophagus; BMI, body mass index; MetS, metabolic syndrome; ERD, erosive reflux disease; GERD, gastroesophageal reflux disease

In univariate logistic regression analyses (Table 2), the risk of BE was significantly associated with central obesity (OR, 2.53; 95% CI, 1.78–3.60), *H*. *pylori* infection (OR, 0.50; 95% CI, 0.34–0.74), metabolic syndrome (OR, 2.02; 95% CI, 1.38–2.96), and BMI (OR, 1.07; 95% CI, 1.02–1.12). In the multivariate-adjusted analysis (Table 3), the association with central obesity (adjusted OR, 2.79; 95% CI, 1.89–4.12) and *H*. *pylori* infection (adjusted OR, 0.46; 95% CI, 0.30–0.70) remained statistically significant, whereas metabolic syndrome and BMI were not independently associated with BE.

**Table 2:** Univariate analysis for risk factors of BE.

Characteristics	BE	
	OR (95% CI)	*p*-value
**Central obesity**	2.53 (1.78–3.61)	<0.001
**H. pylori infection**	0.50 (0.34–0.74)	<0.001
**MetS**	2.02 (1.38–2.96)	0.003
**BMI**	1.07 (1.02–1.12)	0.008
**Obesity**	1.33 (0.89–1.99)	0.160
**Drinking**	1.23 (0.86–1.77)	0.249
**Smoking**	0.94 (0.64–1.38)	0.757

BE, Barrett's esophagus; OR, odds ratio; BMI, body mass index; MetS, metabolic syndrome

**Table 3:** Multi-variate analysis for risk factors of BE.

Characteristics	BE	
	OR (95% CI)	*p*-value
**Central obesity**	2.79 (1.89–4.12)	<0.001
***H*. *pylori* infection**	0.46 (0.30–0.70)	<0.001

BE, Barrett's esophagus; OR, odds ratio

## Discussion

This study took a standardized approach to clarify risk factors of BE in an ethnic-specific population. Our results support that central obesity defined by waist circumference, rather than BMI or other indicators of body adiposity, plays a key role in ethnic Chinese. Besides, *H*. *pylori* infection is inversely associated with BE in this population. This finding may have imperative public health implications to this part of world where the prevalence of *H*. *pylori* infection remains high.

The relationship between Barrett’s esophagus and obesity has been extensively studied, but the results were contradictory[19–24]. Besides, they were mainly case-control studies from western countries. In 2009, a meta-analysis which summarized 11 studies found an increased risk of BE (OR, 1.4) among white patients with BMI ≥ 30kg/m^2^[25].

Taking body fat distribution into consideration, recent studies from Corley et al.[23] and Akiyama et al.[26] further demonstrated that abdominal obesity (measured by waist circumference or abdominal CT scan), instead of total obesity (measured by BMI[27]), was more strongly associated with BE, on the other hand, gluteofemoral obesity(measured by hip circumference) carries the protective effect[28]. Our analysis revealed this correlation also existed in Chinese population, suggesting that the risk of BE may be mediated through increased abdominal fat, which is associated with increased intragastric pressure[29]. Increased gastroesophageal pressure gradient may contribute to more transient lower esophageal sphincter relaxation with acid reflux, thus increased the risk of BE[30]. In addition to mechanical effects, abdominal fat is also associated with alteration in the adipose-related hormones, which may play a role in the pathogenesis of BE[31].

The inversed association between BE and *H*. *pylori* infection has been reported by Corley et al.[32] in a case-control study. And they found the association may be partially mediated through acid reflux. Fischbach et al. [33] further demonstrated that the decreased risk of BE in *H*. *pylori*-infected patients may be attributed to lowered gastric acidity, due to corpus atrophy or use of antisecretory drugs. Antisecretory drug effect has been minimized in our study since we had excluded PPI users in BE group. Besides, PPI was not allowed in our control group (without erosive esophagitis) as well according to Taiwan’s health insurance system. Our results were the firstly reported Chinese data regarding the inverse association between BE and *H*. *pylori* infection, in an area where the *H*. *pylori* prevalence was almost twice as high as previously reported in Western countries [32, 33]. There are 6 Asian studies that explored the relationship between *H*. *pylori* infection and BE[10, 11, 34–37]. When pooling together[3], *H pylori* infection was not associated with histologic BE. But these studies were less powered than our work in BE case numbers. In the most similar study protocol as ours by Rajendra et al.[34], those subjects with peptic ulcer disease were excluded at initial enrollment. The effect of *H*. *pylori* infection may thus be underestimated.

There are several strengths in our analysis. First, this is a large Barrett’s cohort in Chinese ethnics, and we use only patients with an incident diagnosis of BE, thereby minimizing selection bias. Second, our healthy controls all underwent endoscopy exam and excluded erosive esophagitis, which has been a strong confounder both in *H*. *pylori* infection and obesity. Third, the data were collected prospectively with a systemic protocol.

Some potential limitations still existed in our work. First, the case-control design cannot make further causal inference. Second, there was no control with reflux esophagitis in our study, thus making it difficult to compare with other similar risk-factor studies. Dore et. al [38] recently reported that the protective effect of *H*. *pylori* against BE may be secondary to GERD in a Caucasian population. Because our control group did not consist of patients with reflux esophagitis, it is beyond the scope of the present study to elucidate the role of GERD in the association between Barrett’s esophagus and *H*.*pylori* infection. More epidemiologic data would be required to test the independent effect of GERD in Chinese population.

In summary, our study bridges the knowledge gap in ethnic Chinese population that the risk of BE is associated with increased central obesity (rather than BMI) but inversely associated with *H*.*pylori* infection.
